# Cuproptosis‐Related Ferroptosis Gene Signature: A Prognostic Tool for Colon Cancer Patients

**DOI:** 10.1002/cnr2.70372

**Published:** 2025-10-28

**Authors:** Yanlin Tan, Jinxiu Zhang, Ruoxi Cheng, Wenfang Yang, Xiaoping Pan, Kaoyan Feng, Mengbin Qin, Jie'an Huang

**Affiliations:** ^1^ Department of Gastroenterology The Second Affiliated Hospital of Guangxi Medical University Nanning China

**Keywords:** colon cancer, cuproptosis, ferroptosis, prognosis, tumor microenvironment landscape

## Abstract

**Background:**

Ferroptosis and cuprotosis, two distinct mechanisms of programmed cell death, play key roles in colon cancer development. This study aimed to construct a prognostic model for predicting colon adenocarcinoma (COAD) prognosis based on the differential expression of cuproptosis‐related ferroptosis genes (CFRGs).

**Methods and Results:**

Transcriptomic data and clinical data of COAD patients were obtained from The Cancer Genome Atlas and Gene Expression Omnibus databases. A combination of methods, including analysis of variance, Pearson correlation analysis, Least absolute shrinkage and selection operator (LASSO) algorithm, and Cox regression was used to construct the CFRGs signatures. In addition, multiple algorithmic strategies were employed to explore the potential association between risk scores and immune infiltration features. Single‐cell datasets were used to analyze model genes. Somatic mutations and drug sensitivities were compared across risk groups. Immunohistochemical analysis was performed to verify the expression of the main characterized genes. We identified eight pivotal genes in constructing the CFRGs signature. Single‐cell RNA sequencing revealed differential expression of CFRGs in the COAD tumor microenvironment. The nomogram confirmed that risk scoring serves as an independent prognostic factor for COAD. The high‐risk group exhibited higher immune cell and stromal cell infiltration, as well as immune checkpoint expression. Patients in the high‐risk group may benefit from olanzapine administration. Both RT‐PCR and immunohistochemistry have confirmed that the expression levels of GLS and YAP1 in COAD tissues are significantly higher than those in adjacent non‐cancerous tissues.

**Conclusions:**

The CFRGs risk prognosis model can effectively predict patients' immune infiltration and immunotherapy response, providing a new reference basis for individualized treatment plans for COAD patients.

## 1 Introduction

Colorectal cancer (CRC) ranks among the top three malignant tumors globally, in terms of both incidence and mortality. According to the latest global statistics spanning 2014–2025, CRC accounted for the highest proportion (54.3%) of all early‐onset gastrointestinal (GI) cancers worldwide, with its incidence demonstrating a consistent year‐on‐year increase [1]. The rise in colon cancer incidence in the United States may be attributed to a variety of modifiable risk factors, such as smoking, unhealthy eating habits, excessive alcohol consumption, and obesity [2]. The ongoing advancements in treatment modalities, including refinements in precision surgery and targeted therapy, have helped increase the 5‐year survival rate of colon adenocarcinoma (COAD) patients to 65%. However, patients with advanced disease and distant metastases continue to have a dismal prognosis [3]. An increasing amount of research supports the application of multigene signatures to enhance risk stratification and predict prognosis in patients with CRC [4, 5]. Therefore, identifying more sensitive molecular markers is crucial for accurately predicting prognosis and monitoring treatment efficacy in patients with colon cancer.

Recent years have witnessed the elucidation of several mechanisms of cell death, which have, in turn, inspired novel therapeutic strategies for malignant tumors. Ferroptosis is a unique and non‐apoptotic form of iron‐dependent cell death, representing one of the key pathways of regulated cell death (RCD) triggered by iron‐dependent lipid peroxidation in the cell membrane. Its core driving mechanism lies in the inhibition of the cystine‐glutamate antiporter. This inhibitory process leads to a decrease in glutathione (GSH) synthesis and the inactivation of glutathione peroxidase 4 (GPX4), ultimately resulting in cell death due to excessive lipid peroxidation [6, 7]. In recent years, there has been evidence suggesting that ferroptosis plays a significant role in regulating the tumor microenvironment (TME). For instance, in glioblastoma research, tumor‐infiltrating neutrophils/PMN‐MDSCs can promote ferroptosis in tumor cells, thereby inhibiting tumor growth [8]. Overexpression of ANO1 in gastrointestinal cancer cells inhibits ferroptosis of tumor cells, promotes the recruitment of cancer‐associated fibroblasts (CAFs) by cancer cells, confers resistance to immunotherapy, and thereby affects the therapeutic effect of cancer treatment [9]. Numerous genes have been implicated in the regulation of ferroptosis. *NAT10*‐mediated acetylation of N4‐acetocytidine (ac4C) has been shown to upregulate the expression of *FSP1*, a protein that inhibits ferroptosis, thereby promoting colon cancer growth and metastasis [10]. The oncogene LINC01606 has been identified as establishing a positive feedback mechanism with the Wnt/β‐catenin signaling pathway, which results in the suppression of ferroptosis and enhanced chemotactic properties of tumor stem cells, making it a potential therapeutic target for colon cancer [11].

The cuproptosis pathway refers to the process where excessive copper ions within cells are transported to mitochondria via ionophores and reduced to copper (I) by FDX1. The increased copper (I) content binds to acylated components in the tricarboxylic acid (TCA) cycle, triggering the aggregation of acylated proteins, destabilization of iron‐sulfur cluster proteins, leading to proteotoxic stress and ultimately resulting in cell death [12]. Latest scientific findings have revealed the significance of cuprotosis in the progression of various diseases, including malignant tumors, cardiovascular diseases, metabolic syndrome, and neurodegenerative disorders [13, 14, 15, 16]. Notably, elevated serum copper ion levels have been closely linked to a higher incidence and unfavorable clinical outcomes in individuals diagnosed with lung cancer [17] and CRC [18]. Recent studies have demonstrated that the cuproptosis‐related gene FDX1 exhibits low expression in COAD and is associated with prognosis. Its overexpression can inhibit the growth and metastasis of COAD by suppressing the progression of epithelial‐mesenchymal transition (EMT), showing promise as a potential therapeutic target [19]. Notably, KRAS‐mutant CRC can establish a copper tolerance mechanism by upregulating ATP7A and promoting macropinocytosis, thereby evading cuproptosis. However, copper chelators (such as TTM) or ATP7A inhibition can reverse this mechanism, selectively inducing cuproptosis in KRAS‐mutant cells and significantly suppressing tumor growth, representing a potential precision therapeutic strategy for KRAS‐mutant CRC [20]. In addition, cuproptosis also plays a crucial role in regulating the TME. In CRC, the combination of copper ionophores and nanotechnology enables efficient copper delivery to induce cuproptosis. This process not only directly kills tumor cells but also reprograms CAFs and the immune microenvironment, making cuproptosis an immunogenic cell death (ICD) trigger and providing a novel therapeutic paradigm for CRC treatment based on cuproptosis [21].

The interaction between copper and iron homeostasis plays a role in numerous physiological and pathological processes (including cancer progression). Previous studies have confirmed that cuproptosis and ferroptosis do not exist independently but are mutually regulated through oxidative stress, GSH metabolism, metal ion homeostasis, and immune regulation, affecting tumor occurrence, development, and treatment. Research shows that cuproptosis inhibits the activity of CAFs, and ferroptosis inhibits the expression of GPX4 or ACSL4, jointly weakening the tumor‐promoting functions of the TME [7]. Excess copper leads to the depletion of Fe‐S cluster proteins in a manner reliant on FDX1, consequently reducing the suppressive impact of mitochondrial proteins on ferroptosis [22]. However, it is still unclear whether the genetic characteristics related to cuproptosis and ferroptosis can be used for diagnostic purposes, to assess drug sensitivity, and to predict the immune treatment response of COAD. Therefore, a deeper understanding of these mechanisms is helpful for developing more effective anti‐tumor treatment strategies.

The evidence presented above indicates that the interaction between copper and iron balance is involved in various physiological and pathophysiological mechanisms, such as the advancement of cancer. The regulatory mechanisms governing cuproptosis and ferroptosis are anticipated to emerge as potential therapeutic targets for colon cancer. However, it remains unclear whether the genetic signatures associated with cuproptosis and ferroptosis can be utilized for diagnostic purposes, assessing drug sensitivity, and predicting immunotherapeutic responses in colon cancer.

This study utilized an innovative method that involved the integration and analysis of data sourced from the Cancer Genome Atlas (TCGA) COAD dataset alongside the Gene Expression Omnibus (GEO) database. This comprehensive analysis enabled us to elucidate the characteristics of cuproptosis‐related ferroptosis genes (CFRGs). This research not only deepens the comprehension of cuproptosis and ferroptosis by establishing a connection between the two but also highlights possible therapeutic targets for combating colon cancer. Our findings reveal that distinct CFRGs‐associated subtypes exhibit distinctive immune cell infiltration (ICI) characteristics. Eight differentially expressed CFRGs were ultimately identified, and a proportional hazards regression model was constructed based on this signature. Leveraging this model, we analyzed ICI, tumor mutation load (TMB), drug sensitivity, and immune checkpoints to explore the potential mechanisms of CFRGs in colon cancer. The findings of this study provide a new theoretical basis for predicting the prognosis of COAD patients and guiding individualized immunotherapy strategies.

## 2 Materials and Methods

### 2.1 Data Resources and Preprocessing

The essential information for this research was obtained from the TCGA (https://portal.cancer.gov/). The dataset comprised RNA‐seq data for 524 COAD patients, accompanied by relevant clinical data, as well as single nucleotide variation (SNV) data for 428 tumors and copy number variation (CNV) data for 506 tumors. Additionally, the GSE39582 and single‐cell dataset GSE166555 were obtained from the GEO database (https://www.ncbi.nlm.nih.gov/geo/). Furthermore, the single‐cell dataset GSE166555 was downloaded for COAD. For subsequent analysis, the RNA sequencing data obtained from the TCGA and GEO databases were consistently transformed into log2 per million transcripts (TPM) data. A comprehensive list of 464 genes associated with ferroptosis was acquired from the FerrDb database (http://www.zhounan.org/FerrDb/current/), alongside 19 genes linked to cuproptosis that were gathered from existing literature [23, 24].

### 2.2 Identification of Differentially Expressed Genes (DEGs) Linked to COAD Prognosis

DEGs associated with ferroptosis and cuprotosis were detected utilizing the “limma” R package. Heatmaps and volcano plots were created utilizing the “pheatmap” and “ggplot2” R packages, respectively. Univariate Cox analysis was employed to identify DEGs with prognostic value. Additionally, a correlation network graph was constructed for the prognostic‐related DEGs, and the String database (https://cn.string‐db.org/) was utilized to conduct protein‐protein interaction (PPI) analysis. The waterfall plot visualized the results of somatic mutation data analysis. Furthermore, chromosome circle plots were used to analyze CNVs of prognosis‐related DEGs.

### 2.3 Unsupervised Clustering Analysis of Genes Associated With Prognosis

Using the R package “ConsensusClusterPlus”, we conducted an unsupervised cluster analysis on 181 CFRGs to detect distinct molecular clusters. The cumulative distribution function (CDF) and delta region maps were employed to ascertain the optimal cluster count. In addition, heat maps of prognostically relevant DEGs in conjunction with clinicopathological features were generated using the “pheatmap” package. Kaplan‐Meier survival curves were plotted to compare the overall survival (OS) differences between the identified subtypes.

### 2.4 Functional Enrichment Analysis

A genome‐wide analysis of gene set variation (GSVA) was utilized to explore the biological pathways associated with various subtypes of CFRGs. To investigate the pathways linked to the two identified isoforms, Gene Ontology (GO) analysis and Kyoto Encyclopedia of Genes and Genomes (KEGG) enrichment analysis were executed. Additionally, gene set enrichment analysis (GSEA) was conducted using the “clusterProfiler” R package to assess differences among the risk groups. The criteria for significant enrichment were defined as follows: absolute standardized enrichment score (NES) > 1, *p* < 0.05, and false discovery rate (FDR) < 0.25.

### 2.5 Tumor Immune Correlation and Tumor Microenvironment Analysis

Single‐sample gene set enrichment analysis (ssGSEA) was performed using the R packages “GSVA” and “GSEABase” to evaluate and measure the infiltration of immune cells and the expression of immune function across samples that represent different subtypes and risk categories. Subsequently, Spearman correlation analysis was used to investigate the connection between CFRG scores and eight genes related to prognosis that are associated with immune cells. The “corrplot” R package was used to visualize correlations between the abundance of various subtypes and distinct immune cells. The “estimate” R package was utilized to conduct TME analysis for both groups classified as high risk and low risk. Additionally, the immunophenotype score (IPS) was used to predict immunotherapy sensitivity.

### 2.6 Development of a Prognostic Signature Associated With Cuproptosis and Ferroptosis

A cohort of 1034 patients from TCGA‐GEO was randomly split into training and validation cohorts in a 1:1 ratio through the “caret” R package. Least absolute shrinkage and selection operator (LASSO)‐Cox regression analysis was performed using the “glmnet” R package. Furthermore, multivariable Cox analysis was performed to pinpoint candidate genes that displayed significant prognostic relevance. The risk score was computed using the equation: Risk score = ∑NI = 1(coefi × Expi). Based on the median risk score, patients were classified into two groups: high‐risk and low‐risk. Afterwards, Kaplan‐Meier curves were created to compare OS between these two risk categories. A receiver operating characteristic curve (ROC) was generated, and the “timeROC” R package was employed to assess the predictive accuracy of the model. To estimate survival probabilities at 1, 3, and 5 years after diagnosis, clinical features were incorporated into the model. Additionally, nomograms and their corresponding calibration plots were developed using the “rms” R package. The effectiveness of the array plots was evaluated using the time‐dependent concordance index (time C‐index).

### 2.7 Tumor Mutation Burden (TMB) Analysis

The complete count of mutations for each COAD sample was obtained and analyzed with a Perl script. The visualization of somatic mutation data was achieved through the use of the “maftools” R package, which produced waterfall maps to aid in interpretation. A Spearman analysis indicated a relationship between the risk score and TMB. To further investigate the genetic landscape, a comparative analysis of CNV profiles was conducted for DEGs associated with prognosis. The results were visualized using chromosome circle plots. Concurrently, RNA dry score analyzes were conducted for risk scores.

### 2.8 Drug Sensitivity Prediction

To explore how high‐risk and low‐risk patients respond to different medications, we leveraged the Cancer Drug Sensitivity Genomics (GDSC) database (https://www.cancerrxgene.org/). Using the R package “pRRophetic”, we estimated the half‐maximal inhibitory concentrations (IC50) for multiple chemotherapeutic agents.

### 2.9 Analysis of Single‐Cell RNA Sequencing Data

Sequencing data were processed using the “Seurat” R package, and low viability cells were removed. Following quality control based on cellular markers, mitochondrial, and ribosomal gene expression, the t‐SNE downscaling algorithm classified the cells into 12 major clusters. Cell types were validated using specific gene markers, and the expression levels of CFRGs were analyzed across different clusters.

### 2.10 Real‐Time Quantitative Polymerase Chain Reaction (RT‐qPCR)

Total RNA was extracted from para‐cancerous tissues and CRC tissues collected at the Second Affiliated Hospital of Guangxi Medical University using the EaStepSuper kit. Subsequently, the concentration and purity of the extracted RNA were assessed utilizing a NanoDrop2000 spectrophotometer. After the assessment, the PrimeScript RT kit was employed to reverse‐transcribe the extracted total RNA into complementary deoxyribonucleic acid (cDNA). Next, the expression levels of target mRNAs were detected using the SYBR Green kit, with the housekeeping gene GAPDH serving as a quantitative reference. During data analysis, the 2−∆∆Ct method was applied to normalize the detection results. The information on the qPCR primers used in this experiment is detailed in Table 1.

**TABLE 1:** RT‐PCR primer sequence.

Primer	Sequence (5′ → 3′)	
GLS	Forward Reverse	GGGTATGATGTGCTGGTCTCC TGAGTGAAGAAAGGTCCAAGC
YAP1	Forward Reverse	CAACTCCAACCAGCAGCAAC AGGGCTAACTCCTGCCGAA
GAPDH	Forward Reverse	GCACCGTCAAGGCTGAGA CCTGCAAATGAGCCCCAGC

### 2.11 Immunohistochemistry

Thirty‐five cases of colon cancer tissues and twenty‐four cases of paracancerous tissues were collected from the Second Affiliated Hospital of Guangxi Medical University for immunohistochemical staining. This study has been reviewed and approved by the Ethics Committee of the Second Affiliated Hospital of Guangxi Medical University. First, the tissues were subjected to fixation, dehydration, embedding, and other procedures to prepare paraffin sections. Subsequently, the sections were dewaxed and rehydrated to restore their antigenicity, followed by heat‐induced antigen retrieval using a citrate buffer (pH 6.0). Endogenous peroxidase activity in the tissues was blocked with 3% hydrogen peroxide (H_2_O_2_). After washing three times with phosphate‐buffered saline (PBS), the sections were blocked with 5% bovine serum albumin (BSA) for 20 min. The sections were then incubated overnight with primary antibodies against *GLS* (HUABIO, China, 1:200) and *YAP1* (HUABIO, China, 1:200) at 4°C. Subsequently, secondary antibodies were applied using the PV‐9000 kit (ZSGB‐BIO, China) for 25 min. Antibody staining was performed using the DAB reagent. Immunostained images were captured using a microscope and analyzed using Image J software. The intensity of IHC staining was evaluated using the average optical density (AOD) score, and these data were used for subsequent statistical analysis.

### 2.12 Statistical Analysis

Data analysis and statistical evaluations were carried out using R software (version 4.3.2) in conjunction with Strawberry Perl Software (version 5.38.2.2). To compare two groups, the Wilcoxon rank‐sum test was employed, whereas analysis of variance (ANOVA) facilitated comparisons among multiple groups. *p* values less than 0.05 were deemed to indicate statistical significance.

## 3 Results

### 3.1 Analysis of Differential Expression, Prognostic Value, and Genetic Variation Characteristics of CFRGs

**FIGURE 1:**
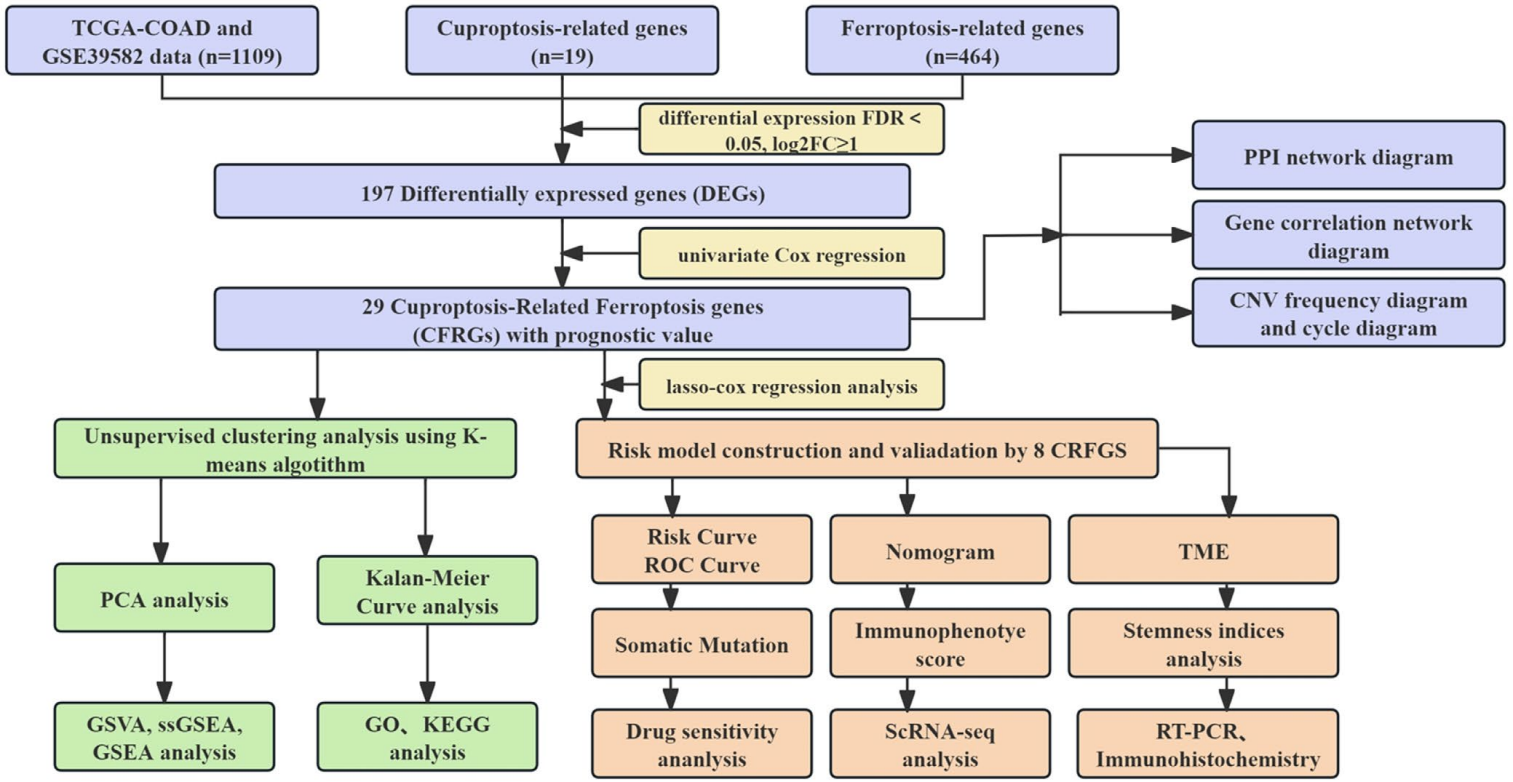
Study design and methodology flowchart. The flowchart illustrates the step‐by‐step approach used in this study, from data collection and preprocessing to model construction and validation.

**FIGURE 2:**
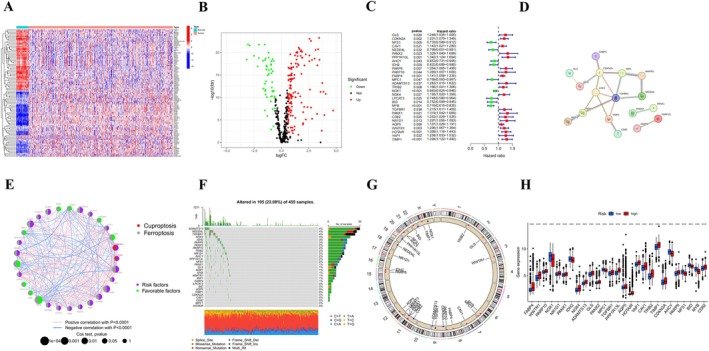
Identification of CFRGs in COAD and their genetic and transcriptional changes. (A, B) Differential expression of CFRGs in COAD; (C) Forest maps of 29 CFRGs associated with prognosis. Among them, 20 genes are negatively correlated with prognosis, and the remaining 9 genes show better prognosis; (D, E) Network diagram of interactions between the 29 genes. The size of the circles represents the *p*‐value of each gene for survival prognosis. Red represents risk factors, and green dots represent favorable factors. The thickness of the lines represents the correlation value between genes. Red lines and blue lines represent positive and negative correlations regulated by genes, respectively; (F) CNV mutations across the 29 CFRGs; (G) Chromosomal distribution of CNV mutations in the CFRGs; (H) mRNA expression differences of the 29 CFRGs between normal and tumor samples. ***p < 0.001.

The workflow of the study is depicted in Figure 1. A summary of the clinicopathological features of patients from the TCGA‐COAD and GSE39582 datasets can be found in Table 2. Based on literature reports, we selected 464 FRGs and 19 CRGs for further analysis, as listed in Table S1. We performed cluster analysis of the 197 DEGs identified and generated heat maps and volcano maps (Figure 2A,B; Table S2). To evaluate the prognostic importance of the DEGs, we merged the overall OS data from 1037 patients sourced from TCGA‐COAD and GSE39582 (see Table S3), identifying a total of 29 CFRGs with a significance level of *p* < 0.05. Of these, 20 genes were recognized as risk factors, whereas the other nine genes were classified as protective factors (hazard ratio < 1) (Figure 2C). To elucidate the complex relationships between CFRGs, we constructed network diagrams. The PPI network diagram (interaction score ≥ 0.40) revealed an interaction between *GLS* proteins and *IDH2* proteins (Figure 2D,E). Furthermore, our CNV frequency analysis detected widespread CNV mutations in 29 regulatory factors (Figure 2F). The chromosome ring map demonstrated the location of genes in the chromosome ring (Figure 2G). Additionally, differential expression analysis of the 29 CFRGs across different risk groups revealed significant differences (Figure 2H).

**TABLE 2:** The clinical characteristics of patients in the TCGA and GSE39582 dataset.

Variable	Number of samples
Age at diagnosis	
≤ 65	378
> 65	573
Gender	
Male	509
Female	442
Stage	
I	104
II	421
III	302
IV	124
T	
T1	21
T2	115
T3	652
T4	163
M	
M0	823
M1	125
MX	3
N	
N0	546
N1	228
N2	171
N3	6

### 3.2 Identification of Molecular Subtypes of COAD Based on 29 CFRGs and Characteristics of the Immune Microenvironment

**FIGURE 3:**
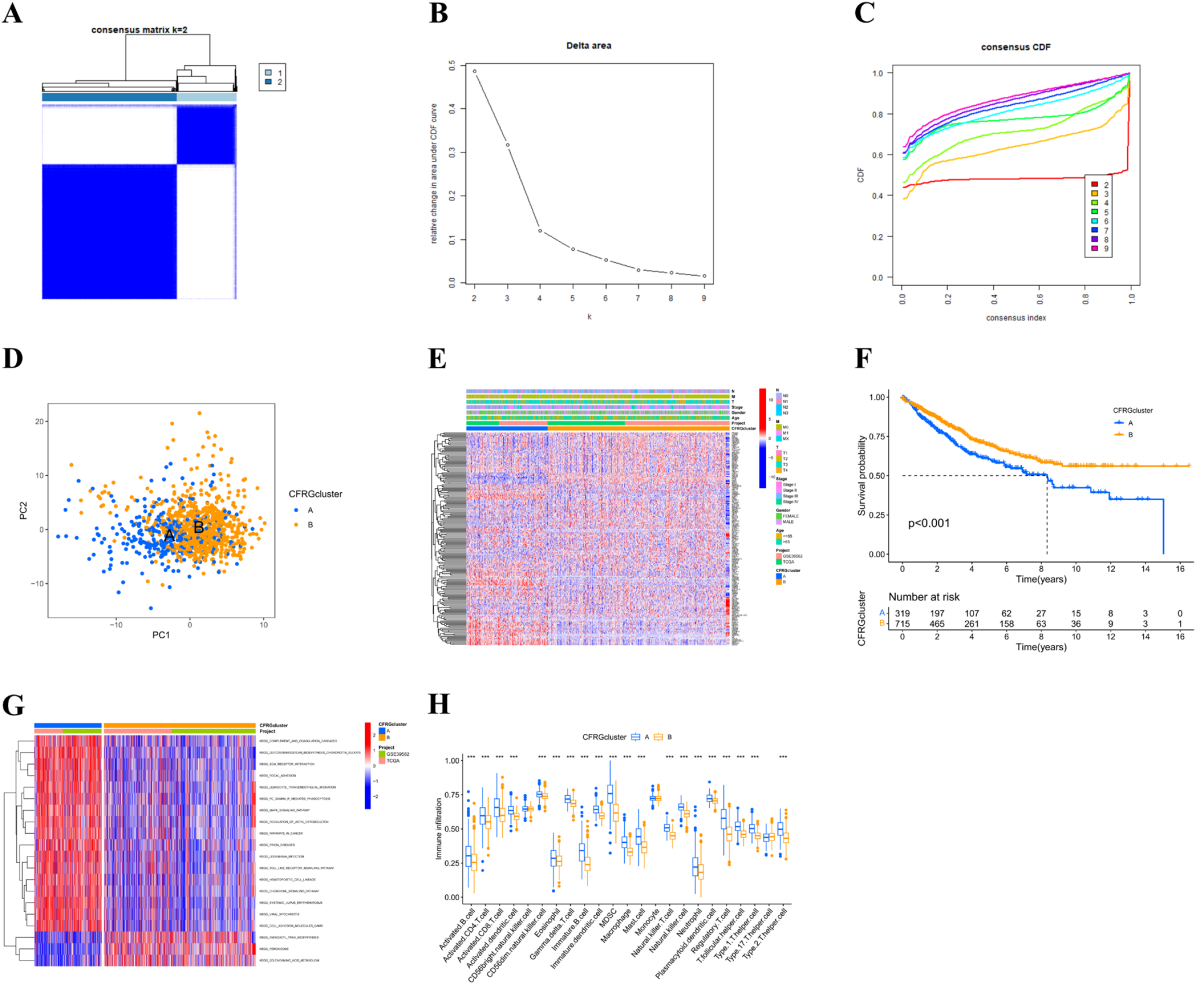
Identification of CFRGs subtypes and their immunological characteristics in COAD. (A) Consensus clustering analysis identifies two distinct CFRG subtypes in COAD. (B) CDF curve graph and (C) Delta Area Plot. When *K* = 4, the slope of the CDF significantly decreases and the area reaches an approximate maximum, indicating that the clustering result is relatively stable at this time; (D) PCA of the two identified clusters; (E) Heatmap of prognostic DEGs combined with subtype‐specific clinical features; (F) The Kaplan‐Meier curves for predicting the survival prognosis of these two subtypes of patients; (G) GSVA is used to evaluate the biological functional differences between the two subtypes; (H) Comparison of immune cell infiltration patterns between the two clusters. ***p < 0.001.

Unsupervised cluster analysis of the 29 CFRGs indicated that the best clustering outcome was obtained with *k* = 2. Therefore, we classified patients from two cohorts (TCGA‐COAD, GSE39582; *n* = 1043) into 2 subtypes (Figure 3A–C). The principal component analysis (PCA) demonstrated notable distinctions between these two subtypes, as shown in Figure 3D. A heatmap illustrating the relationship among CFRG clustering, gene expression, and various clinical characteristics (Figure 3E). The Kaplan‐Meier survival analysis indicated that individuals with subtype B demonstrated a considerably improved prognosis in comparison to those with subtype A (*p* < 0.001) (Figure 3F). Next, we performed GSVA‐enriched pathway analysis to determine the biological functions of the two subtypes. This analysis revealed that subtype B was mainly associated with biological pathways involved in amino acid biosynthesis and oxidative stress, compared to subtype A (Figure 3G). ssGSEA analysis unexpectedly showed that most activated immune cells and innate immune cells were significantly enriched in subtype A, suggesting higher immune infiltration and related functions in this subtype (Figure 3H).

### 3.3 Enrichment Analysis of CFRGs Reveals Their Involvement in Key Pathways of Immune Regulation and Tumor Progression

**FIGURE 4:**
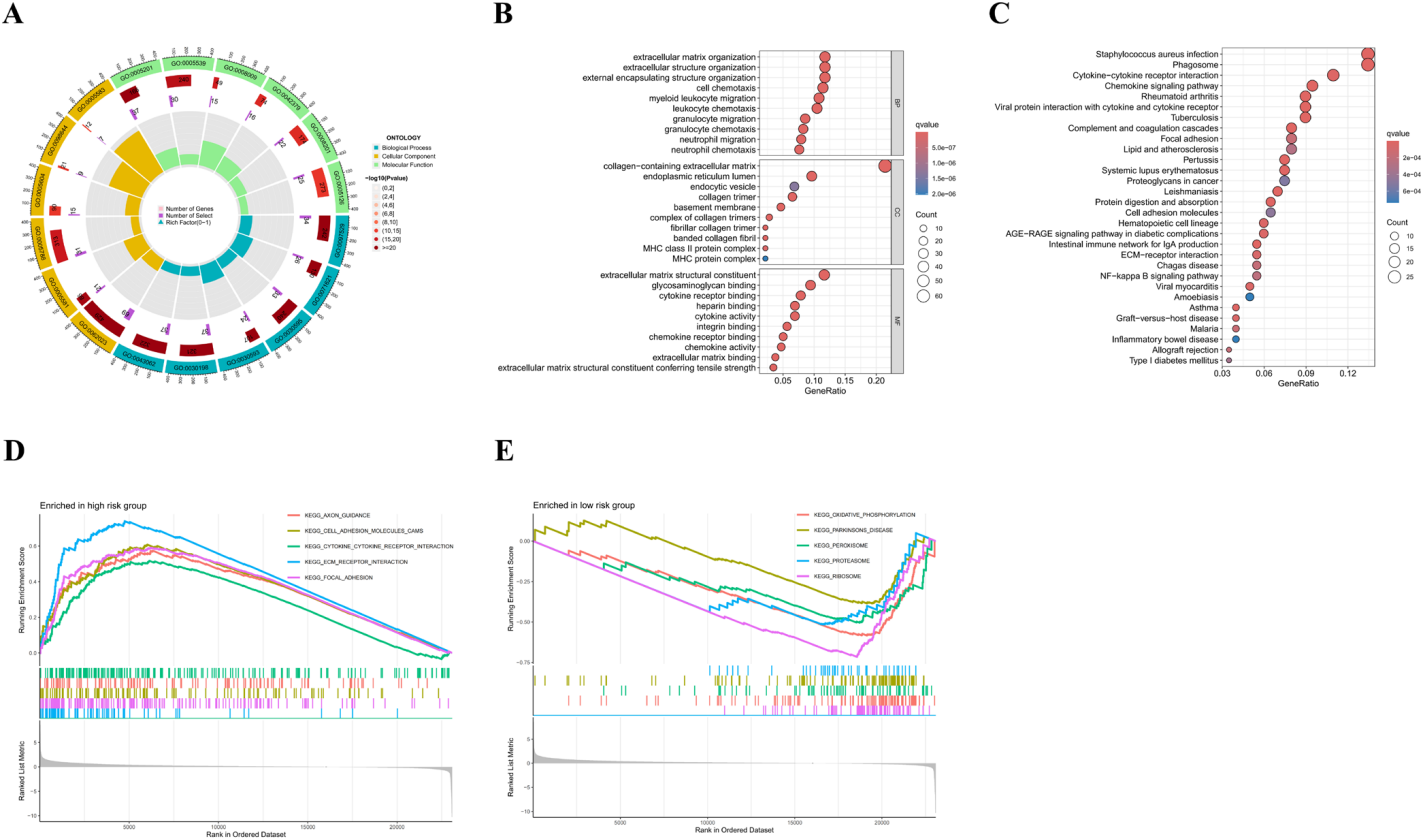
Biomolecular function enrichment analysis was conducted for the differentially expressed genes in the two subtypes. GO (A) and KEGG (B, C) analyzes of DEGs in CFRG subtypes; (D, E) GSEA comparing high‐ and low‐risk cohorts; GO, Gene Ontology; KEGG, Kyoto Encyclopedia of Genes and Genomes; GSEA, gene collection enrichment analysis.

Analysis of functional enrichment indicated that the genes identified were mainly linked to leukocyte movement and chemotaxis, the composition and maintenance of the extracellular matrix (ECM), as well as the regulation of the major histocompatibility complex (MHC) protein family (Figure 4A–C). GSEA results, as shown in Figure 4D, indicated that multiple pathways related to cancer progression, including migration, proliferation, and differentiation, were enriched in the high‐risk group. These pathways encompassed interactions between cytokines and receptors, as well as those between ECM receptors and focal adhesions (FAs). Metabolic pathways such as protein synthesis and degradation, oxidative phosphorylation, and fatty acid metabolism showed greater enrichment in the low‐risk group (Figure 4E). The detailed results of GSEA are provided in Table S4. These observations indicate that biological pathways are more dynamically active in the high‐risk group, closely associated with the progression of cancer.

### 3.4 Prognostic Risk Model for COAD Constructed Based on Eight CFRGs and Multi‐Cohort Validation

**FIGURE 5:**
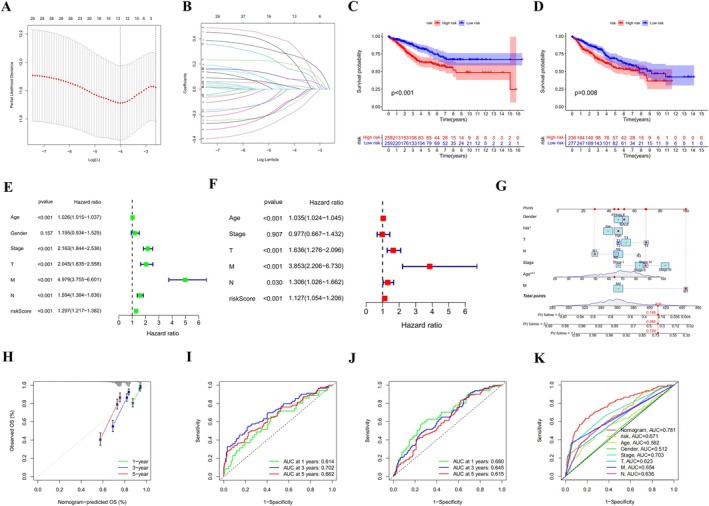
Construction and validation of a risk scoring model for predicting overall survival in patients with COAD. (A, B) LASSO method for identifying CFRGs associated with prognosis. The optimal penalty parameter (λ) value was selected. 13 genes were screened out at the point where the cross‐validation error was the lowest; survival analysis of the high‐ and low‐risk groups in the training cohort (C) and validation cohort (D); univariate (E) and multivariate (F) Cox regression analysis, and multivariate COX regression analysis were conducted on the 13 genes obtained previously to screen out eight genes; (G) nomogram predicting the survival rate of patients; (H) the calibration curve indicates that the 1‐year, 3‐year OS results predicted by the nomogram are in good agreement with the actual OS results; time‐dependent ROC curves for predicting prognosis in the validation cohort (I) and training cohort (J); (K) ROC curve evaluating the performance of the risk model combined with clinical data. *p < 0.05; ***p < 0.001.

To assess the prognostic relevance of the CFRGs feature model, samples obtained from the TCGA and GEO databases were combined and randomly divided into two separate groups: the training cohort and the validation cohort. A summary of the clinical characteristics for these cohorts can be found in Table S5. Following this, a prognostic risk model was developed utilizing the eight CFRGs, informed by the optimal penalty parameter (*λ*) values derived from LASSO‐Cox regression analysis (Figure 5A,B). The Kaplan‐Meier curves for the training cohort (Figure 5C) and the validation cohort (Figure 5D) demonstrated significant variances between the high‐risk and low‐risk groups. This finding indicates that patients with elevated risk scores experienced a worse prognosis and reduced OS. These results suggest a satisfactory predictive ability of this risk score‐based prognostic model.

### 3.5 Construction, Calibration Verification, and Predictive Performance of a Survival Prediction Nomogram for COAD Patients

To further assess the model's independent predictive capacity, Cox regression analysis was conducted. The findings indicated that age, TNM stage, and risk score served as independent prognostic predictors (Figure 5E,F). Given that risk scores are clinically insufficient to predict OS in COAD patients, we created a nomogram utilizing a risk score based on CFRGs (Figure 5G). The column line plots showed that the nomogram exhibited reliable calibration and precision (Figure 5H). For the validation cohort, the area under the ROC curve (AUC) values for 1‐, 3‐, and 5‐year survival were 0.680, 0.645, and 0.615, respectively, while for the training cohort, the corresponding AUCs were 0.614, 0.702, and 0.662 (Figure 5I,J). Notably, the nomogram (ROC = 0.781) demonstrated superior predictive capability compared to using the risk score and staging score alone (Figure 5K).

### 3.6 High‐Risk Score of CFRGs Is Related to Immunosuppressive Microenvironment and Immune Escape Characteristics

**FIGURE 6:**
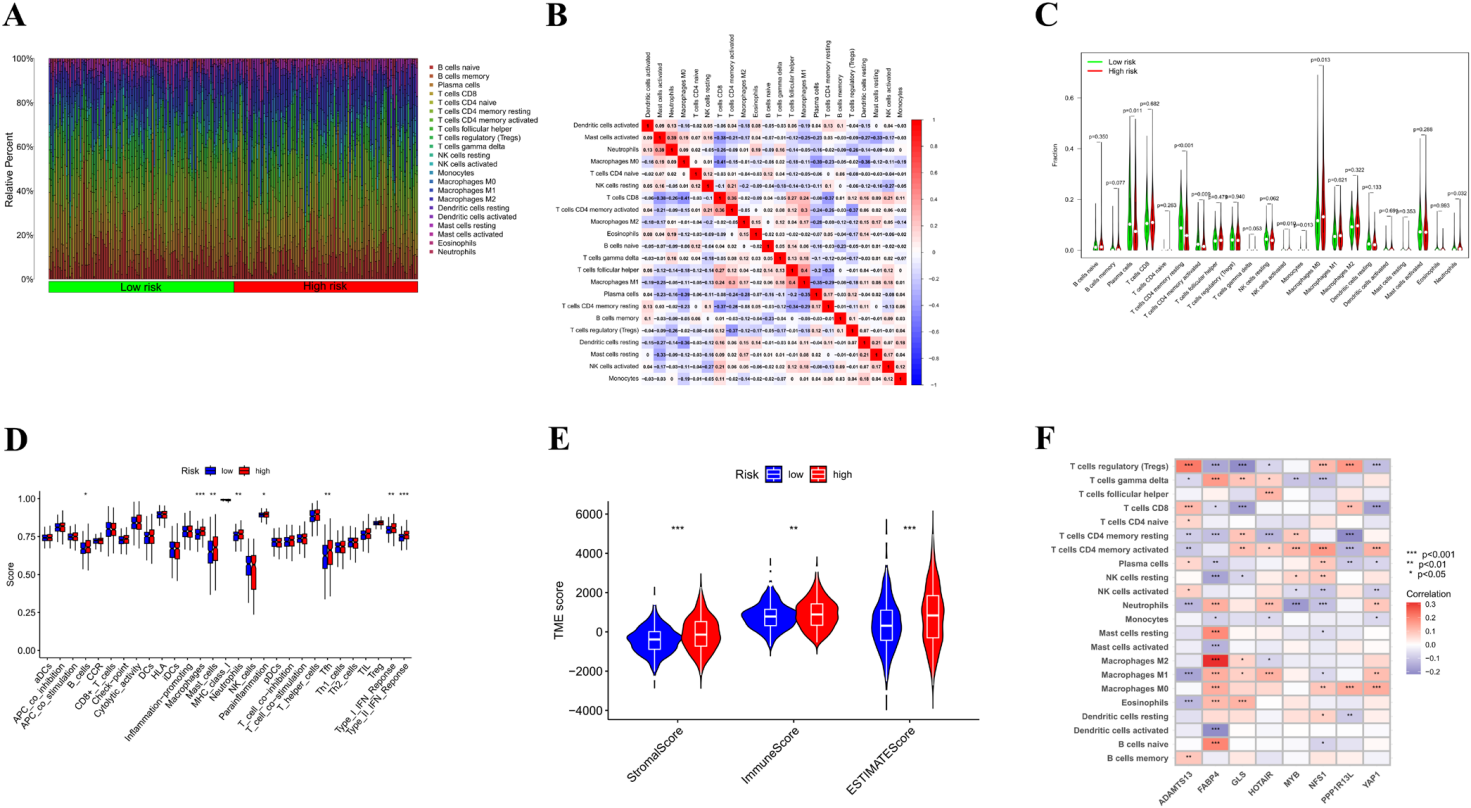
Analysis of the characteristics of the tumor microenvironment between the high‐risk group and the low‐risk group based on the CFRGs score. (A) The abundance ratio of immune cells in the high‐risk group and the low‐risk group obtained using the CIBERSORT algorithm; (B) Correlation analysis of immune cells in different risk groups; (C) Functional analysis of immune cells in high‐ and low‐risk groups; (D) Correlation analysis between immune cell infiltration and risk score; (E) TME analysis comparing high‐ and low‐risk groups; (F) CIBERSORT algorithm analysis revealing the correlation between CFRGs and immune cell infiltration. *p < 0.05; **p < 0.01; ***p < 0.001.

Stacked histograms illustrated the distribution of 22 types of immune cells across different samples (Figure 6A). The interrelationships among the infiltration levels of these 22 immune cells are depicted in Figure 6B. Patients categorized in the high‐risk group showed elevated infiltration of activated NK cells, monocytes, M0 macrophages, and neutrophils, while those in the low‐risk group had increased infiltration of plasma cells, resting memory CD4+ T cells, and other immune cell types (Figure 6C). Furthermore, immune cells that play roles in antigen presentation and tumor killing, including B cells, macrophages, mast cells, and neutrophils, scored higher in the high‐risk group (Figure 6D). Notably, our observations indicated that the high‐risk group exhibited significantly elevated scores for immunity, stroma, and ESTIMATE when compared with the low‐risk group. This suggests that individuals classified in the high‐risk category are more susceptible to tumor invasion and metastasis (Figure 6E). We also found varying degrees of correlation between the eight model genes and immune cell populations (Figure 6F).

### 3.7 High‐Risk Score of CFRGs Is Associated With Increased Tumor Mutation Burden (TMB) and Poor Prognosis

**FIGURE 7:**
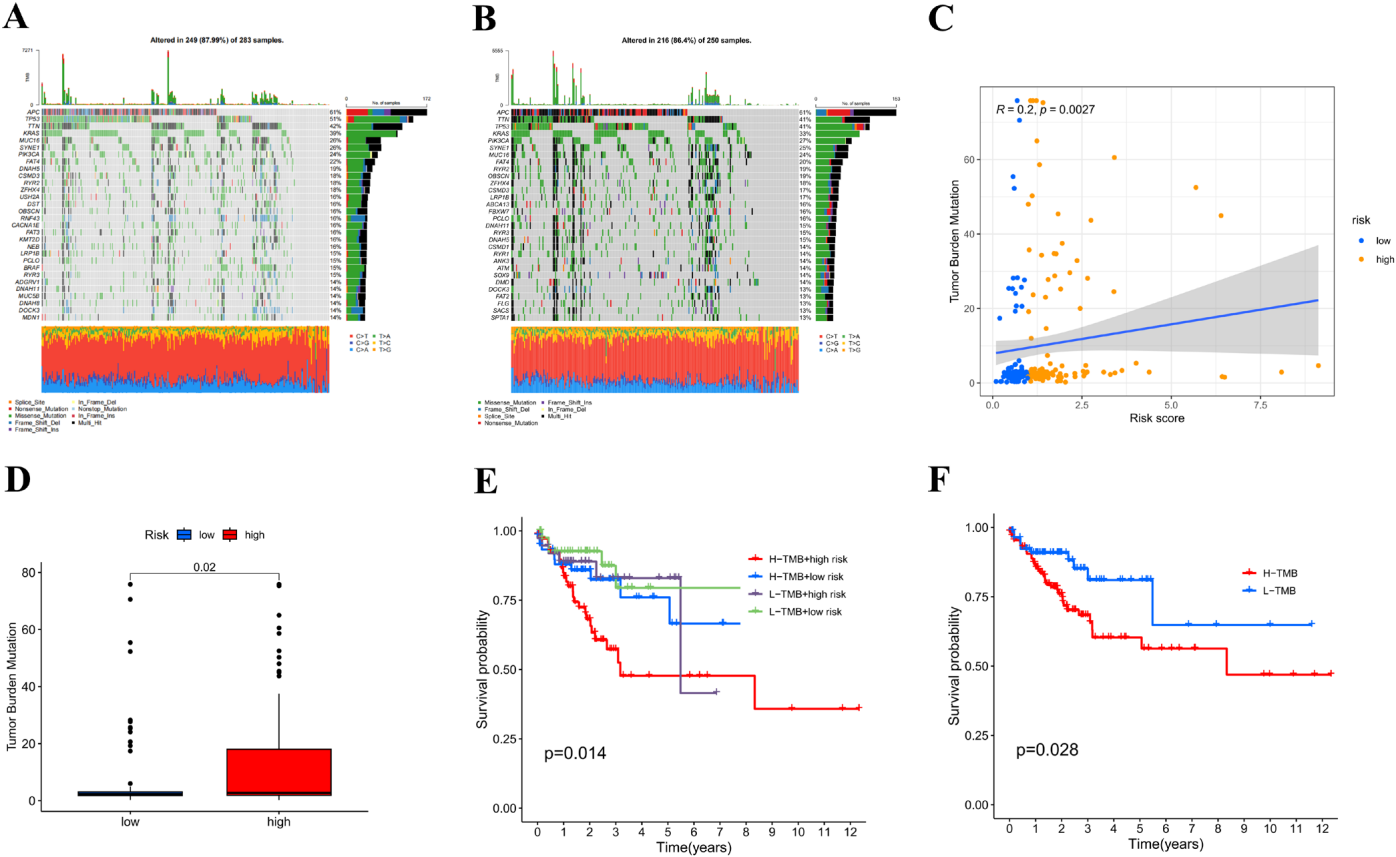
To explore the relationship between the CFRGs risk score and TMB, as well as the somatic mutation burden. (A, B) Waterfall plots illustrating the top 20 genes with the highest mutation frequency in the high‐ and low‐risk groups; (C) Correlation of TMB and CFRGs risk score; (D) Comparison of TMB differences between the high‐ and low‐risk groups; (E) The Kaplan‐Meier survival curves between different risk groups based on mRNA risk and TMB; (F) Kaplan‐Meier curves based on high and low TMB.

In order to examine the distribution of somatic mutations between the high‐ and low‐risk cohorts, we generated waterfall plots for both risk‐score categories. Genes including *APC, TP53, TTN, KRAS, MUC16, PIK3CA, SYNE1*, and *FAT4* exhibited the highest frequencies of mutations across all samples. Furthermore, multiple mutations and missense mutations were the most commonly observed mutation types (Figure 7A,B). Spearman's correlation analysis revealed a notable positive correlation between TMB and risk score (*R* = 0.2, *p* = 0.0027; Figure 7C). Analysis of variance revealed that TMB was considerably elevated in the high‐risk group compared to the low‐risk group (*p* = 0.02; Figure 7D). Of note, it is important to highlight that patients exhibiting both low TMB and low‐risk scores showed markedly improved survival rates (Figure 7E,F).

### 3.8 Patients With Low‐Risk Score of CFRGs Are More Sensitive to Immunotherapy and Chemotherapy Drugs

**FIGURE 8:**
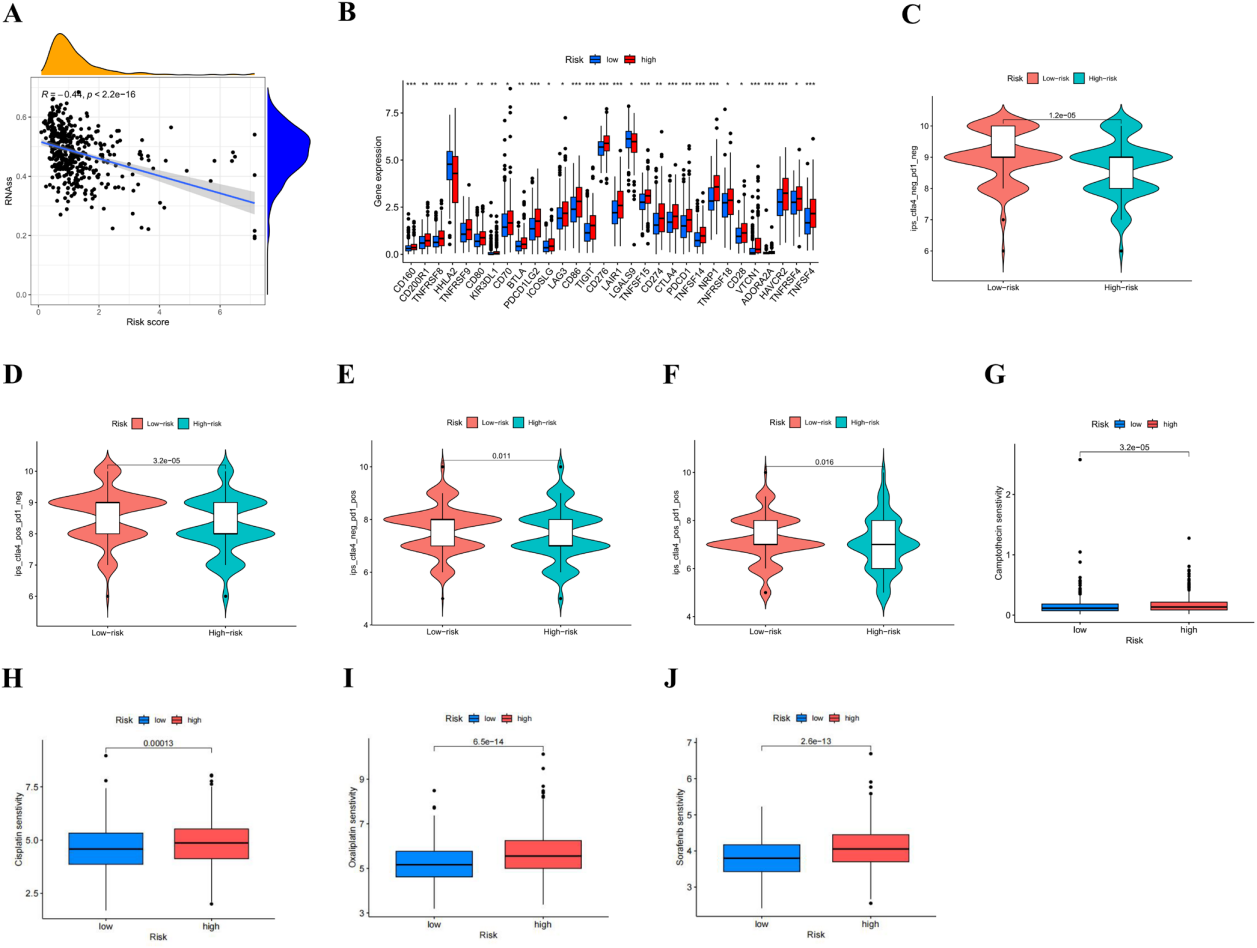
Based on the correlation analysis between the CFRGs risk score and the response to immunotherapy. (A) Relationship between the cancer stem cell (CSC) index and the CFRGs risk score; (B) Differential expression analysis of immune checkpoints between high‐ and low‐risk groups; (C–F) Analysis of the differences in the response to CTLA‐4 and PD‐1 immune checkpoint inhibitor therapy between patients in the high‐risk group and the low‐risk group; (G–J) Differences in IC50 between high‐ and low‐risk groups and CRC‐targeted drugs. *p < 0.05; **p < 0.01; ***p < 0.001.

We further observed a negative correlation between stem cell markers and risk score (*R* = −0.44, *p* < 0.001), suggesting that patients with lower risk scores exhibited more evident stem cell characteristics (Figure 8A). We found that the expression levels of most immune checkpoint genes, such as *CTLA4* and *PDCD1*, were considerably elevated in the high‐risk group in contrast to the low‐risk group (Figure 8B). IPS prediction revealed higher IPS values for *CTLA4*+/*PD1*−, *CTLA4*−/*PD1*+, and *CTLA4*+/*PD1* treatments in the low‐risk group, suggesting that the low‐risk group responded more favorably to *CTLA4* and *PD‐1* mono‐ and combination therapies (Figure 8C–F). As shown in Figure 8G–J, the analysis of IC50 value differences between the two risk groups showed that the IC50 of camptothecin, cisplatin, oxaliplatin, and sorafenib were lower in the low‐risk group. These results suggest that individuals in the low‐risk category may exhibit greater sensitivity to tumor‐targeted therapies.

### 3.9 Single‐Cell Level Reveals the Cell‐Specific Expression of CFRGs in the Tumor Microenvironment of COAD

**FIGURE 9:**
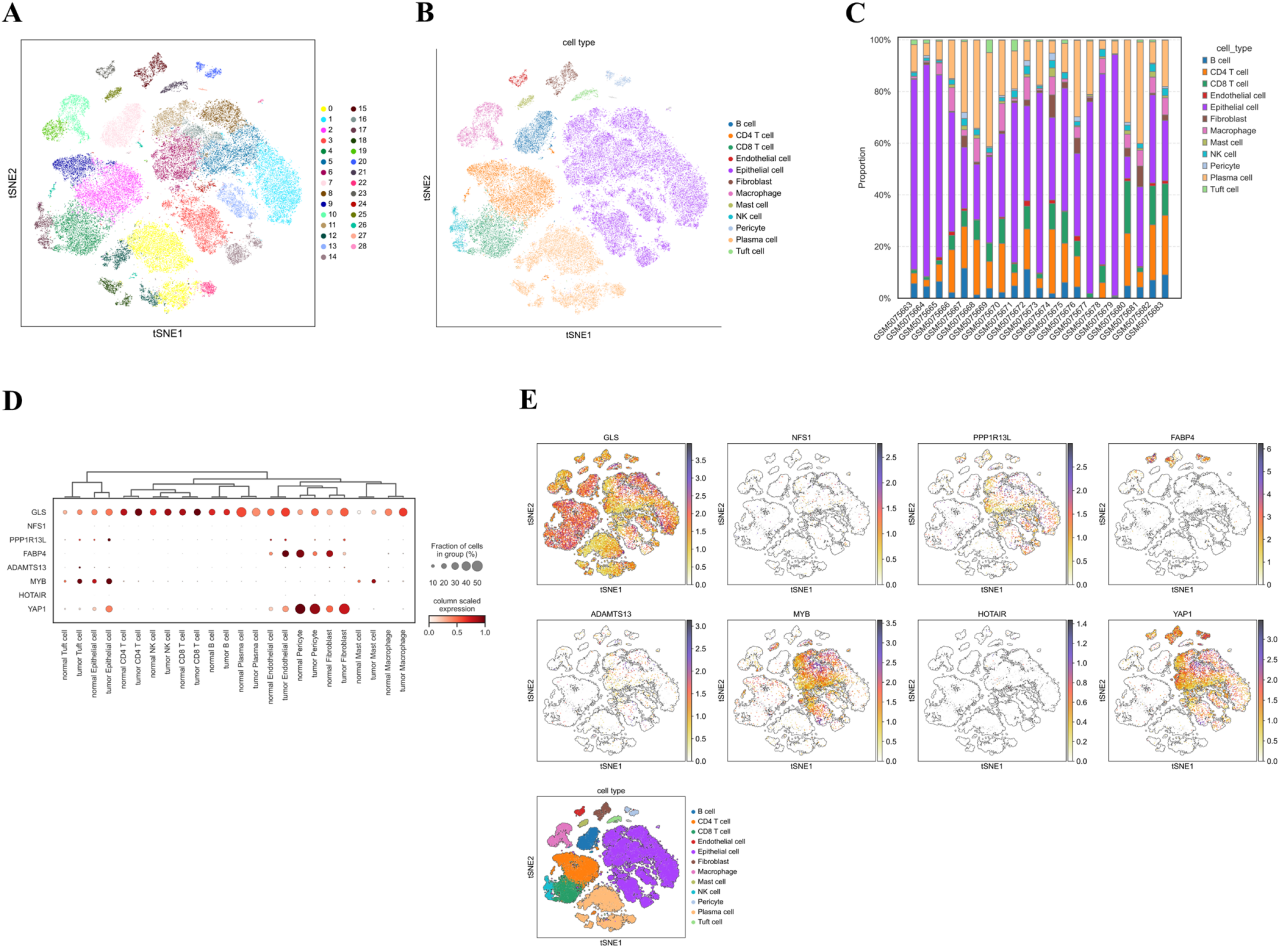
Single‐cell RNA sequencing profiling of tumor and normal tissues. (A) t‐Distributed Stochastic Neighbor Embedding (t‐SNE) analysis annotating each cluster; (B) Identification of cell types within each cell population; (C) Stacked histogram illustrating the proportion of cell types in each sample; (D, E) Bubble plots showing the expression levels and distribution of the eight CFRGs.

We investigated the expression patterns of CFRGs within colon cancer tissues using single‐cell resolution analysis of RNA transcriptome data. To ensure data quality, we excluded cells with gene assay counts exceeding 5000 or below 200 per cell. t‐Distributed Stochastic Neighbor Embedding (t‐SNE) visualization revealed 28 distinct cell clusters (Figure 9A). We identified and annotated 12 immune cell subtypes (Figure 9B). Bar graphs illustrate the percentage of each cell type present in the various samples (Figure 9C). We visualized the expression of the eight characterized genes by generating bubble plots. As shown in Figure 9D,E, *GLS* exhibited high expression across almost all cell subtypes. In contrast, *YAP1* and *FABP4* displayed increased expression mainly in pericytes and fibroblasts. Additionally, *PPP1R13L*, *MYB*, and *YAP1* showed high expression in epithelial cells.

### 3.10 Clinical Validation of Key Genes GLS and YAP1 of the CFRGs Model

**FIGURE 10:**
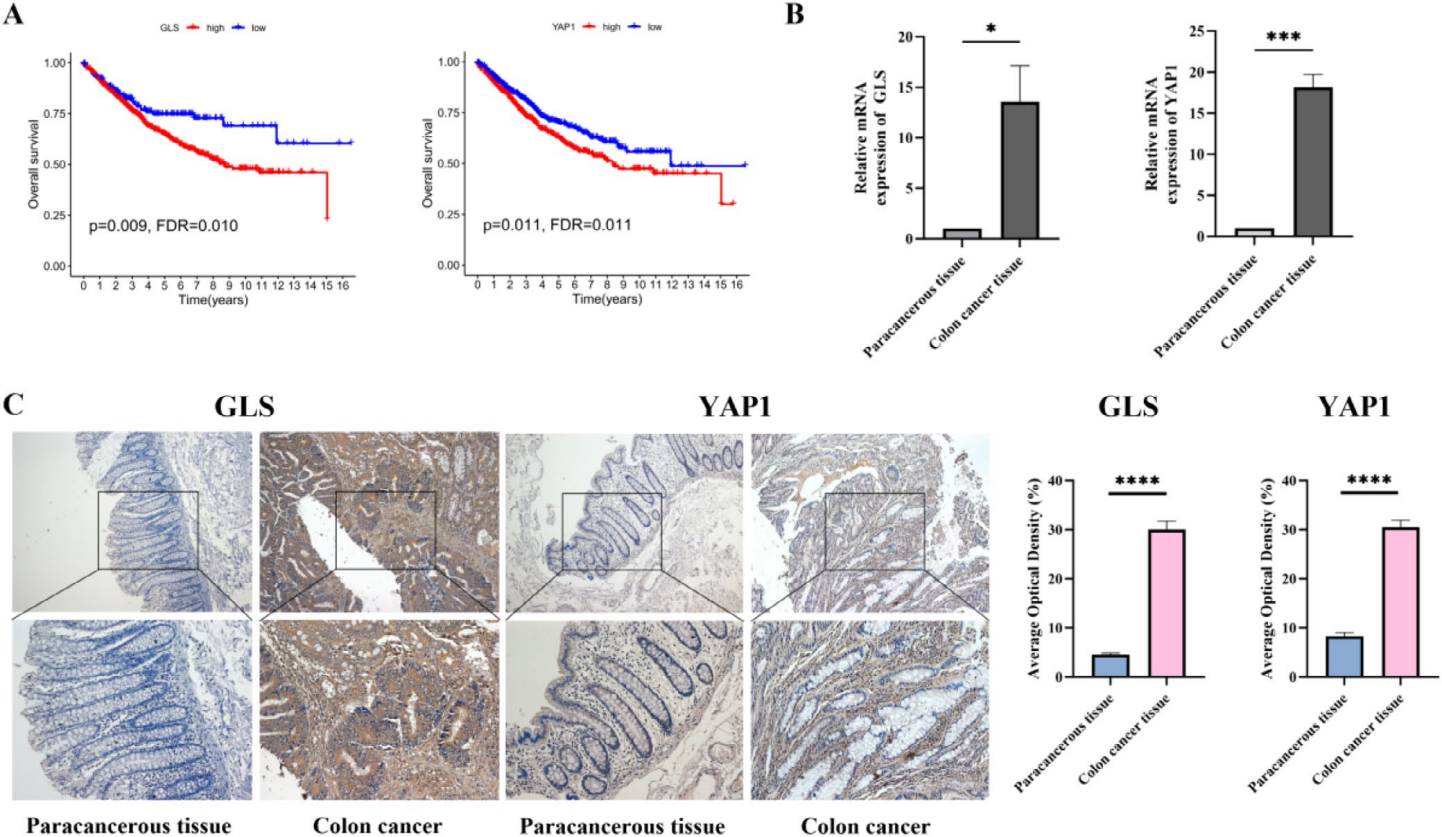
RT‐PCR and IHC validation of key genes. (A) Relationship between the expression levels of *GLS* and *YAP1* in the TCGA database and the prognosis of COAD patients; (B) The mRNA expression of *GLS* and *YAP1* in paracancerous tissue and COAD tissues; (C) Representative immunohistochemical staining images of *GLS* and *YAP1* in adjacent tissues and COAD tumor tissues. *p < 0.05; ***p < 0.001; ****p < 0.0001.

*GLS and YAP1 represent key genes associated with cuproptosis and ferroptosis, respectively*. Both *GLS* and *YAP1* exhibited high correlation coefficients. Moreover, the overexpression of *GLS* and *YAP1* was significantly linked to unfavorable outcomes in patients with (Figure 10A). Gene association analysis and PPI also indicated a potential association between *GLS* and *YAP1*. To further validate our research findings, we employed RT‐PCR technology to conduct detection and analysis on the adjacent tissue and COAD tissue. The results showed that, compared with the adjacent tissue, both the *GLS* and *YAP1* genes exhibited significant overexpression characteristics in the COAD tissue (Figure 10B). Additionally, through immunohistochemical staining analysis of paraffin sections from COAD patients, we discovered that the expression levels of *GLS* and *YAP1* proteins in colon cancer tissues were also significantly upregulated. This result was highly consistent with our previous analysis conclusion (Figure 10C).

## 4 Discussion

RCD, encompassing apoptosis, ferroptosis, and cuproptosis, plays a pivotal role in the initiation, progression, and treatment of cancer. A profound understanding of the molecular mechanisms underlying RCD not only facilitates the discovery of novel mechanisms in carcinogenesis but also provides innovative strategies for developing targeted therapeutic agents [25]. Numerous studies have confirmed the involvement of individual genes related to copper‐dependent apoptosis and iron‐dependent apoptosis in the progression of malignant tumors [26, 27, 28]. At present, numerous studies have focused separately on the roles of single regulatory genes for cuproptosis or ferroptosis in tumors. Nonetheless, there have been no studies exploring the clinical prognosis of COAD by simultaneously combining multiple CRGs and FRGs. This research sought to investigate the robust relationship between CFRG characteristics and the prognosis of COAD, providing novel insights for innovative cancer therapies.

Considering that colon cancer exhibits distinct genetic features and a considerable level of heterogeneity among its molecular subtypes, this variability directly results in notable differences in patient prognosis across various subtypes. Hence, it is crucial to investigate new prognostic biomarkers or develop corresponding models to support clinical diagnosis and treatment. In this research, we combined the TCGA‐COAD and GSE166555 cohorts to elucidate the mutational landscape, chromosomal location, and protein interactions of CFRGs in CRGs and FRGs in COAD through comprehensive multi‐omics analysis. Based on CFRGs expression profiles, COAD patients were stratified into two distinct molecular subtypes, which demonstrated marked disparities in clinical characteristics, TME composition, and ICI patterns. The CFRGs model demonstrated excellent performance in predicting prognosis and immunotherapeutic susceptibility. Moreover, it facilitated the screening of potential immune drugs for patients in high‐ and low‐risk groups, highlighting its substantial potential for clinical application.

To accurately assess cuproptosis and ferroptosis patterns in COAD patients, we constructed predictive models based on eight CFRGs genes (*GLS, NFS1, PPP1R13L, FABP4, ADAMTS13, MYB, HOTAIR*, and *YAP1*). The research found that the inhibition of *SIRT4* would activate *GLS*, thereby initiating *AKT* activation, suggesting that *GLS* might influence the proliferation, migration, and invasion of colon cancer cells through the *AKT/GSK3β/CyclinD1* pathway under the regulation of *SIRT4* [29]. *GLS1* regulates redox homeostasis (*Nrf2/ROS*) and autophagy pathways to drive the proliferation, migration, and survival of CRC cells [30]. The abnormal high expression of *GLS* is closely related to the cisplatin resistance phenotype of colon cancer cells, and inhibiting *GLS1* can work in synergy with cisplatin to effectively induce the death of colon cancer cells, indicating that *GLS* may become a potential intervention target for improving the chemotherapy effect of colon cancer [31]. In this study, the high expression of *GLS* in COAD is closely related to the high‐risk score, poor prognosis, and positive correlation with anti‐tumor infiltration.

In lung adenocarcinoma, high expression of *NFS1* has been shown to maintain iron–sulfur cluster cofactors in cancer cells, thereby resisting oxidative damage and avoiding ferroptosis [32]. The expression of *PPP1R3L* has been found to be negatively regulated by the down‐regulation of *miR‐124*, promoting the malignant progression of glioblastoma [33]. *FABP4*, a fatty acid‐binding protein, has been shown to inhibit *CADM3* transcription by regulating *PPAR‐γ*, thereby promoting gastric cancer metastasis [34]. Deficiency of the metalloproteinase *ADAMTS13* has been linked to an increased risk of thromboembolism in patients with CRC, resulting in lower OS [35]. The transcription factor *c‐Myb* exerts a tumor‐suppressive function in CRC through the modulation of anti‐tumor immune responses and transcriptional regulation of immune‐related genes [36]. The transcription factor *c‐Myb* exerts a tumor‐suppressive function in CRC through the modulation of anti‐tumor immune responses and transcriptional regulation of immune‐related genes [37].

Previous studies have confirmed that *YAP1* may partially reverse the inhibitory effect of *SNTB1* knockdown on the phenotype of CRC cells and the *Wnt/β‐catenin/MYC* signaling pathway, thereby affecting the invasiveness of colon cancer cells and the growth and metastasis of tumors in vivo [38]. As the core effector molecule of the *GABABR1‐Hippo* pathway that inhibits CRC metastasis, the activation of *YAP1* can strongly drive the proliferation, migration, and invasion of CRC cells by inducing EMT [38]. *YAP1* modulates CRC progression and metastasis through the regulation of oncogenic signaling cascades, cytokine networks, and suppression of M2‐type tumor‐associated macrophage (TAM) polarization [39]. In this study, we found that *YAP1* was overexpressed in COAD and was associated with M1‐type macrophages, as well as being correlated with a poorer prognosis.

As one of the most abundant stromal components of the TME, CAFs play a key role in cancer progression due to their complex heterogeneity [40]. Recent studies have highlighted the adaptability and dual functionality of CAFs in oncology, positioning them as significant prognostic indicators and promising targets for cancer therapies [40, 41]. In prostate cancer models, pharmacological inhibition of *YAP1* was shown to induce phenotypic switching of CAFs from tumor‐promoting to tumor‐suppressive states, consequently enhancing the responsiveness of prostate tumors to immune checkpoint blockade (ICB) therapy [42]. In this study, scRNA‐seq results revealed that the expressions of *GLS*, *PPP1R13L*, *MYB*, and *YAP1* were significantly elevated in tumor epithelial cells. *GLS* and *YAP1* were also expressed at high levels in CAFs. Moreover, RT‐PCR and immunohistochemical analyzes confirmed that the expression levels of *GLS* and *YAP1* were significantly upregulated in colon cancer tissues. These results provided a theoretical basis for subsequent studies on potential therapeutic targets for CRC (such as *GLS* and *YAP1*).

The TME and the molecules it secretes are essential in the initiation and progression of tumors, thus regarded as possible targets for cancer treatment [43]. Comparative profiling of high‐ versus low‐risk cohorts demonstrated elevated infiltration of B lymphocytes, macrophages, neutrophils, and T follicular helper (Tfh) cells in the high‐risk subgroup. Existing research suggests that targeting the B cell–Tfh cell–IL‐21 axis can enhance specific anti‐tumor immune responses [44]. Macrophages, as central cells in cancer, are also involved in cancer formation, from initiation to distant metastasis [45]. As critical mediators within the TME, TAMs significantly contribute to malignant transformation, neovascularization, metastatic dissemination, and unfavorable clinical outcomes [46]. In addition, cancer cells can trigger the creation of neutrophil extracellular traps (NETs), which in turn can facilitate cancer cell invasion and migration in vitro [47]. These findings contribute to a deeper understanding of the potential mechanisms by which cuproptosis and ferroptosis are involved in immune regulation, providing a theoretical basis for the development of novel immunotherapy strategies for COAD.

TMB and immune checkpoints are key predictors for assessing sensitivity to cancer immunotherapy [48]. It is important to highlight that we found increased expression levels of immune checkpoints, such as *CTLA4*, *PDCD1*, and *CD276*, within the high‐risk group. As a cell surface receptor and a key checkpoint for T cells, *PD‐1* negatively regulates T cell immune activity, promoting immune evasion of cancer [49]. Recent studies have indicated that using anti‐*PD‐1* antibodies in conjunction with other cancer treatment agents, like anti‐*CTLA‐4* antibodies, exhibits initial therapeutic activity in metastatic colorectal cancer (mCRC) patients harboring mismatch repair deficiency (dMMR) or microsatellite instability‐high (MSI‐H) phenotypes, compared to monotherapy. This combination therapy represents a promising treatment strategy, offering improved efficacy and risk control [50]. Therefore, this signature will facilitate further evaluation of the responsiveness of CRC patients to immunotherapy drugs.

Notably, to demonstrate the potential increased effectiveness of immunotherapy in high‐risk populations, we examined the responsiveness of various chemotherapeutic agents among patients categorized into high‐risk and low‐risk groups. The findings indicate that lapatinib and oxaliplatin could offer greater advantages for patients identified as high‐risk according to the CFRG signature. A clinical trial revealed that the use of oxaliplatin alongside other traditional chemotherapy agents, such as 5‐fluorouracil, considerably enhanced the outcomes for individuals with colon cancer following surgery [51]. Consequently, the features of CFRGs may offer personalized treatment alternatives to patients.

In conclusion, this study innovatively constructed an eight‐gene CFRGs prognostic signature model based on the cross‐regulatory mechanisms of cuproptosis and ferroptosis. Not only does it provide a novel and effective tool for prognostic prediction, but it also reveals its potential value in shaping the immune microenvironment, assessing TMB, and predicting treatment responses. The combination of CFRGs may more comprehensively reflect the biological behavior of tumors, enhancing the robustness and clinical applicability of the prognostic model and offering a fresh perspective for predicting the prognosis of COAD patients from a multi‐gene combined viewpoint. However, our study still has certain limitations. We still need more in vitro and in vivo experiments to validate the data based on bioinformatics. Furthermore, primarily based on retrospective analysis of existing data from public databases, it may be susceptible to data bias, and subsequent large‐scale prospective clinical trials are necessary to validate the practicality and robustness of the model.

## Author Contributions

Yanlin Tan, Jinxiu Zhang, Mengbin Qin, and Jie'an Huang conceived and designed the study. Yanlin Tan and Jinxiu Zhang performed the experiments and drafted the paper. Ruoxi Cheng, Wenfang Yang, Xiaoping Pan, and Kaoyan Feng performed data collection and analysis. Yanlin Tan and Jinxiu Zhang were responsible for writing the manuscript. All authors contributed to the article and approved the submitted version.

## Conflicts of Interest

The authors declare no conflicts of interest.

## Supporting information


**Table S1:** The gene list of 19 cuproptosis‐related genes and 464 ferroptosis‐related genes.

**Table S2:** Identification of 197 differentially expressed genes.

**Table S3:**: Overall survival (OS) data of 1037 COAD patients in the TCGA‐COAD and GSE39582 datasets.

**Table S4:** Detailed results of GSEA enrichment analysis.

**Table S5:** Clinical characteristics of samples in the training and test cohort.

## Data Availability

The data that support the findings of this study are available from the corresponding author upon reasonable request.
